# Carotid intima-medial thickness in patients with severe hypertriglyceridemia

**DOI:** 10.1016/j.athplu.2024.04.001

**Published:** 2024-04-21

**Authors:** Maud Ahmad, Brooke A. Kennedy, Surim Son, Adam D. McIntyre, Julieta Lazarte, Jian Wang, Robert A. Hegele

**Affiliations:** a Department of Medicine, Schulich School of Medicine and Dentistry, Western University, London, Ontario, N6A 5B7, Canada; b Robarts Research Institute, Western University, London, Ontario, N6A 5B7, Canada; c Department of Epidemiology and Biostatistics, Western University, London, ON, N6A 5B7, Canada

**Keywords:** Atherosclerosis, Risk factor, Triglycerides, Chylomicrons, Ultrasound, Imaging, Carotid artery, Hypertriglyceridemia, Hyperlipoproteinemia type V

## Abstract

**Background and aims:**

Severe hypertriglyceridemia (HTG), defined as plasma triglyceride (TG) concentration > 10 mmol/L, is relatively uncommon, and its implications for atherosclerotic cardiovascular disease (ASCVD) risk remain somewhat unclear. We evaluated the association between severe HTG and carotid intima-media thickness (IMT), a marker for ASCVD.

**Methods:**

We studied three clinical cohorts: 88 patients with severe HTG (mean TG level 20.6 mmol/L), 271 patients with familial hypercholesterolemia (FH) as a contrast group, and 70 normolipidemic controls. Carotid IMT was measured using standardized ultrasound imaging. Statistical analysis was conducted using one-way analysis of variance (ANOVA) to compare mean IMT values, analysis of covariance (ANCOVA) to adjust for confounding variables, specifically age and sex, as well as Spearman pairwise correlation analysis between variables.

**Results:**

Unadjusted mean carotid IMT was greater in severe HTG and FH groups compared to controls, however, this was no longer significant for severe HTG after adjustment for age and sex. In contrast, adjusted carotid IMT remained significantly different between the FH and control groups.

**Conclusions:**

Our findings suggest that extreme TG elevations in severe HTG patients are not significantly associated with carotid IMT, in contrast to the increased IMT seen in FH patients. These findings add perspective to the complex relationship between severe HTG and ASCVD risk.

## Highlights

• The relationship between atherosclerosis and chylomicronemia is contentious.
• We evaluated carotid intima-media thickness (IMT) in three distinct patient groups.
• Adjusted carotid IMT was not increased in patients with chylomicronemia vs controls.
• In contrast adjusted carotid IMT was significantly increased in FH patients.
• Severe hypertriglyceridemia appears not to be associated with increased carotid IMT.

## 1 Introduction

The prevalence of severe hypertriglyceridemia (HTG), characterized by a plasma triglyceride (TG) concentration >10 mmol/L (>880 mg/dL), is ∼1 in 400 [1]. Severe HTG often indicates the pathological presence of fasting chylomicrons from two main etiologies: familial chylomicronemia syndrome (FCS) and multifactorial chylomicronemia syndrome (MCS) [[2], [3], [4]]. FCS comprises 1–5% of all cases of severe HTG and is a rare recessive condition that is caused by bi-allelic pathogenic variants in one of five canonical genes, namely *LPL, APOA5, APOC2, GPIHBP1* and *LMF1* [2,3]. In contrast, MCS is much more common but is genetically complex and non-Mendelian [[2], [3], [4]]. MCS patients may have either a single heterozygous variant in one of the five canonical FCS genes or an extreme accumulation of multiple single-nucleotide polymorphisms (SNPs) that confer polygenic risk, or both or neither [[2], [3], [4]]. In addition to genetic susceptibility, severe HTG is often precipitated by secondary factors such as obesity, alcohol, uncontrolled diabetes, and metabolic syndrome [5]. Additionally, several medications can raise TG, including exogenous estrogen, retinoids, corticosteroids, thiazide diuretics, and β-blockers, among others [6].

The most pressing complication for patients with severe HTG is acute pancreatitis [5,6], which has an associated mortality rate of up to 20% or more [7]. In contrast, the association of severe HTG with risk of atherosclerotic cardiovascular disease (ASCVD) is less clear [8]. With mild-to-moderate HTG, in the absence of chylomicrons, observational studies demonstrate an association between elevated TG and ASCVD risk [[9], [10], [11], [12]]. For example, the ICARIA study found that cardiovascular risk increased for each HTG stratum and that HTG was independently associated with ASCVD disease [13]. An observational study from the United Kingdom noted increased risk of all-cause and cardiovascular mortality among individuals from a cohort with severe HTG [14]. However, clinical trials of cardiovascular outcomes using TG-lowering agents such as omega-3 fatty acids [15,16], extended-release niacin [17] and fibrates [18] have been equivocal. Only the REDUCE-IT trial demonstrated cardiovascular benefit of icosapent ethyl, but the benefit was not primarily related to TG-lowering [19]. However, all these studies excluded patients with TG > 5.65 mmol/L (> 500 mg/dL), thus eliminating patients with severe HTG [20].

Carotid intima-media thickness (IMT) is a non-invasive test that has been validated for assessing ASCVD risk and is correlated with coronary artery disease and its risk factors [[21], [22], [23]]. In our lipid clinic, we measure carotid IMT data from patients at their first visit before treatment is initiated, and as a result we have established a unique dataset including patients with severe HTG and familial hypercholesterolemia (FH). FH patients are included as a contrast group due to this condition being a well-established risk factor for ASCVD secondary to elevated low-density lipoprotein cholesterol (LDL-C). In this study, we asked whether patients with severe HTG, specifically MCS, have increased carotid IMT compared to control subjects and contrasted the findings with FH patients.

## 2 Methods

### 2.1 Study subjects

Our study included three cohorts: 1) 88 patients with severe HTG, defined as fasting TG > 10 mmol/L and genetically confirmed to have MCS; 2) 271 patients with genetically confirmed heterozygous FH and untreated LDL-C > 5 mmol/L; and 3) 70 normolipidemic control individuals with baseline fasting TG < 1.5 mmol/L and LDL-C < 3.0 mmol/L. Normal control data were collected through convenience sampling from volunteers from our institute and from unrelated healthy spouses accompanying patients to our clinic. All participants were outpatients at London Health Sciences Centre (University Hospital) Lipid Genetics Clinic and all provided informed consent. The study was approved by the Western University Institutional Review Board, protocol number 0379E.

### 2.2 Molecular analyses

All participants in the study were sequenced with LipidSeq, our targeted next-generation sequencing panel to diagnose monogenic or polygenic FH, FCS, MCS and other dyslipidemias. A comprehensive description of how our lab uses LipidSeq and performs genomic analysis has been reported previously [24]. The LipidSeq panel includes the main canonical FH genes *LDLR, APOB,* and *PCKS9* [25] and the canonical FCS genes *LPL, APOA5, APOC2, GPIHBP1* and *LMF1* [4]. It additionally includes genome-wide associated single-nucleotide polymorphisms (SNPs) implicated in elevated LDL-C and TG. These SNPs form the respective polygenic risk scores. High polygenic risk for either LDL-C or TG is defined as a score greater than the 90th percentile, determined from a normolipidemic reference group [24].

### 2.3 Carotid IMT measurement

A single sonographer obtained all images of carotid IMT at the referral visit for each participant utilizing the Philips HD11 ultrasound device and L12-5 transducer (Philips, Markham, Ontario). The posterior wall of each common carotid artery was measured by averaging 10 1-mm segments, starting 10 mm inferior to the bifurcation point. These measurements were analyzed using the QLAB Advanced Quantification software's standard IMT extension. In our experience, this technique demonstrates greater than 99% consistency within the operator and a technical variation coefficient of < 1%. Carotid IMT measurements were recorded as arithmetic means in millimeters from left and right sides and, additionally, equations were used to convert these to age and sex-adjusted percentile scores [21].

### 2.4 Statistical analysis

For demographic data, the distribution of continuous variables was initially examined for normality using the Shapiro-Wilk test, and pairwise comparisons between different groups were performed using the appropriate parametric or non-parametric tests. Categorical variables were analyzed using the chi-square test to identify any statistically significant differences among the categories. One-way analysis of variance (ANOVA) was used to compare the mean and percentile IMT across disease status. When one-way ANOVA showed statistical significance, the Tukey post hoc test was conducted for pairwise comparison. Normality was assessed using a normal quantile-quantile plot. Kruskal Wallis with Dunn's test was conducted for non-normally distributed data. The homogeneity of variance assumption was assessed using the variance ratio and a residual plot. Analysis of covariance (ANCOVA) was performed to compare the mean IMT across disease status after adjusting for age and sex. The least-square means (lsmean) were derived for adjusted means. For ANCOVA, the homogeneity of regression slopes assumption was assessed using an interaction term. Spearman correlation coefficient was used to assess the correlation of mean IMT with TGs and total cholesterol. All analyses were performed with R version 4.2.1. A p-value <0.05 was considered statistically significant.

Statistical power was calculated to determine the sample size necessary to detect a 10% difference in IMT between the controls and HTG groups, with an alpha of 0.05 and a power of 90%. Based on a control group mean IMT of 0.50 ± 0.10 mm, we determined that 84 subjects per group were required to detect this clinically significant difference.

## 3 Results

### 3.1 Demographic features of study subjects

A total of 429 patients were included. Summary statistics and demographic data for the three patient cohorts can be found in Table 1. The mean ± standard deviation (SD) ages for the severe HTG, FH, and normal control groups were 51.8 ± 10.1, 45.1 ± 16.0, and 32.9 ± 11.9 years, respectively. Significant differences were observed in the body mass index (BMI) between the severe HTG cohort and the other cohorts. However, linear regression modeling showed that BMI was not associated with carotid IMT percentile in severe HTG patients. A similar trend was observed for total cholesterol (TC) in the normolipidemic group, which had significantly lower TC than other cohorts. Differences in age and gender distribution were not significant.

**Table 1:** Participant clinical and demographic information.

Characteristics	HTG patients	FH patients	Normal controls	*HTG versus FH patients (p-value)*	*HTG versus Normal controls (p-value)*	*FH versus Normal controls (p-value)*
*Age (years)*	51.8 ± 10.1	45.1 ± 16.0	32.9 ± 11.9	<0.001	<0.001	<0.001
*Number (females)*	88 (21)	271 (148)	70 (38)	<0.001	<0.001	NS (1.00)
*BMI (kg/m*^*2*^*)*	30.5 ± 5.5	26.8 ± 5.9	24.5 ± 4.1	<0.001	<0.001	0.013
*TC (mmol/L)*	9.89 ± 5.76	8.29 ± 1.92	4.43 ± 0.85	NS (0.32)	<0.001	<0.001
*LDL-C (mmol/L)*	ND	6.22 ± 1.93	2.50 ± 0.76	ND	ND	<0.001
*TG (mmol/L)*	20.6 ± 12.3	1.43 ± 0.80	1.09 ± 0.54	<0.001	<0.001	0.009
*Hypertension (%)*^a^	39 (44.8%)	48 (17.9%)	1 (1.56%)	<0.001	<0.001	<0.001
*Diabetes (%)*^b^	28 (31.8%)	21 (11.4%)	0 (0%)	<0.001	<0.001	0.019

Abbreviations: BMI, body mass index; FH, familial hypercholesterolemia cohort; HTG, severe hypertriglyceridemia cohort; LDL-C, low density lipoprotein cholesterol; TC, total cholesterol; TG, triglycerides; ND, not determined; NS, not significant.

All values reported as mean ± standard deviation, except for adjusted IMT, which are reported as mean ± standard error.

a Hypertension data are missing for 1, 3, and 6 patients in SHTG, FH, and Normal control groups respectively.

b Diabetes data are missing for 5 patients in the Normal control group.

### 3.2 Genetic results

All 88 of the severe HTG patients in our study had MCS. Fourteen had a heterozygous pathogenic variant in one of five canonical FCS genes, namely *LPL, APOA5, APOC2, GPIHBP1* and *LMF1*, 34 had an elevated polygenic score for TG ≥ 90th percentile, 3 had both factors, and 44 had neither. We define FCS as being due to bi-allelic pathogenic variants in one of five canonical genes, namely *LPL, APOA5, APOC2, GPIHBP1* and *LMF1*. By this definition, our study did not include any FCS patients.

Among the FH cohort, 154 had a heterozygous pathogenic variant in *LDLR, APOB,* or *PCSK9* genes, 63 had high an elevated polygenic score for LDL-C ≥ 90th percentile, 22 had both factors, and 71 had neither, but met the Dutch Lipid Clinics Network criteria for likely or definite FH.

### 3.3 Unadjusted analysis

The unadjusted mean carotid IMT values for the three cohorts are shown in Table 2. The mean ± SD carotid IMT for severe HTG, FH, and normal control were 0.62 ± 0.15, 0.61 ± 0.16, and 0.49 ± 0.08 mm, respectively. Pairwise comparisons found that mean carotid IMT between severe HTG and FH was not significantly different (p = 0.74). In contrast, mean carotid IMT was 0.14 mm greater in severe HTG patients than in normal controls (p < 0.001) and 0.12 mm greater in FH patients than in normal controls (p < 0.001).

**Table 2:** Carotid IMT measurements and comparisons between patient and control groups.

Characteristic	HTG patients	FH patients	Normal controls	*HTG versus FH patients (p-value)*	*HTG versus Normal controls (p-value)*	*FH versus Normal controls (p-value)*
*Carotid IMT (mm) (unadjusted)*	0.62 ± 0.15	0.61 ± 0.16	0.49 ± 0.08	NS (0.74)	<0.001	<0.001
*Carotid IMT (mm) (adjusted for age and sex)*	0.56 ± 0.01	0.61 ± 0.01	0.56 ± 0.01	0.007	NS (0.99)	0.010
*Carotid IMT percentile*	55.9 ± 28.0	62.6 ± 27.0	48.0 ± 20.7	0.040	NS (0.12)	<0.001

Abbreviations: FH, familial hypercholesterolemia cohort; HTG, severe hypertriglyceridemia cohort; IMT, intima-media thickness; NS, not significant.

All values reported as mean ± standard deviation, except for adjusted IMT, which are reported as mean ± standard error.

### 3.4 Adjusted analysis by age and sex covariates

Subsequently, the mean carotid IMT adjusted for age and sex was analyzed using analysis of covariance (ANCOVA). The adjusted means ± standard errors of carotid IMT for severe HTG, FH, and normal controls were 0.56 ± 0.01, 0.61 ± 0.01, and 0.56 ± 0.01 mm, respectively, as shown in Table 2. Pairwise comparisons showed that the adjusted mean IMT is significantly lower in severe HTG patients compared to FH patients (p = 0.007). The adjusted mean IMT was significantly higher in FH patients than in normal controls (p = 0.01). There was no significant difference between HTG patients and normal controls (p = 0.99).

### 3.5 Adjusted analysis by carotid IMT percentile

The unadjusted carotid IMT percentiles for the three cohorts are shown in Table 2. The carotid IMT percentile ± SD for severe HTG, FH, and normal controls were 56.0 ± 28.0, 62.6 ± 27.0, and 48.0 ± 20.7 percentiles, respectively. Pairwise comparisons found that the carotid IMT percentile between severe HTG and FH was not significantly different, with a mean difference of −6.60% (p = 0.10). Similarly, there was no significant difference in carotid IMT percentile between severe HTG patients and normal controls, with a mean difference of 7.95% (p = 0.14). In contrast, the carotid IMT percentile was significantly higher in FH patients than in normal controls, with a mean difference of 14.6% (p < 0.001).

## 4 Discussion

In this study, we observed that age- and sex-adjusted mean carotid IMT measurements were not significantly greater in severe HTG patients compared to normal controls. The findings were similar when an independent adjustment was performed using age- and sex-specific percentiles of carotid IMT as the dependent variables. These findings are thus consistent with the idea that patients with severe HTG, predominantly from chylomicrons, do not show significant pre-atherosclerotic changes as indexed by carotid IMT.

Unadjusted carotid IMT at first seemed higher in severe HTG patients compared to normal controls, but this vanished when adjustment for age and sex was included in the statistical model. Carotid IMT increases linearly with age, independent of cardiovascular disease status or other risk factors [21,22]. Indeed, the severe HTG cohort was older than the normal control group (51.4 vs. 32.9 years, p < 0.001). Additionally, carotid IMT is higher in males than females [26]. Again, the severe HTG cohort had a smaller proportion of females than normal controls (24.7 vs 54.2%, p < 0.001). Perhaps unsurprisingly then, analyses adjusting for age and sex found that carotid IMT was not different between severe HTG and normal controls. In contrast, carotid IMT was significantly higher in FH patients compared to the normal controls after adjustment for age and sex, which is consistent with abundant literature supporting the significant risk for ASCVD in FH [27].

A recent consensus statement by the European Atherosclerosis Society described two principal means by which TG levels are associated with ASCVD risk [28]. First, plasma TGs increase risk because they ‘keep bad company’. Individuals with high plasma TG often have several concurrent pro-atherogenic conditions, including obesity, insulin resistance, diabetes, hypertension, and compromised endothelial function [29]. Second, metabolism of TG-rich lipoproteins (TRLs) produces chylomicron remnants, very low-density lipoprotein (VLDL) remnants, and intermediate-density lipoprotein (IDL). Such remnant particles potentially could penetrate the subendothelial space of arterial walls and deposit cholesterol, triggering an inflammatory response plus macrophage recruitment and transformation of macrophages into foam cells. But while such mechanisms seem to apply to mild-to-moderate HTG, the relevance is less clear for patients with severe HTG, in whom chylomicrons themselves are more abundant.

FCS is generally considered as not being associated with increased risk of ASCVD [5]. FCS is characterized by an isolated abundance of chylomicrons and normal to low levels of atherogenic remnants and VLDL because of the major metabolic blockade resulting from dysfunctional lipoprotein lipase [4]. The primary complication these patients face is pancreatitis. In a post hoc analysis, we found 30 patients with severe HTG had a history of acute pancreatitis, of whom 18 had two or more episodes versus zero cases in FH patients and zero cases in normal controls. Elevations in chylomicrons are implicated in the pathophysiology of acute pancreatitis [30], however, these particles are too large to penetrate the arterial wall and are therefore axiomatically non-atherogenic [8]. Consequently, these patients seem not to have increased ASCVD risk. In contrast, MCS patients typically have higher rates of established ASCVD risk factors, such as diabetes, hypertension, smoking, and higher BMI [31]. Indeed, individuals with MCS are at a 2- to 9-fold higher risk of ASCVD [32]. Unlike in FCS, LPL is not completely impaired in patients with MCS, and therefore, these patients also have elevated levels of atherogenic remnant particles. Nevertheless, our results suggest that VLDL and remnant elevations in MCS patients are not substantially influencing ASCVD risk, at least as indexed by carotid IMT.

There are several limitations of our study, including its observational nature. Also, our study focuses on those with severe HTG, particularly MCS. Due to their distinctive abberant lipoprotein metabolism, the results of this study cannot be applied to the wider spectrum spanning mild-to-moderate HTG. Additionally, the cross-sectional nature of this study provides only a single snapshot of risk, using a single imaging modality. Therefore, time-dependent causal relationships cannot be definitively established or followed. Further, our study is limited by our ultrasound technology only measuring IMT, rather than 2-dimensional plaque area and 3-dimensional plaque volume. Future investigations using these imaging modalities would be informative. In addition, we have not considered the effect of mild-to-moderate HTG and thus the effect primarily of VLDL and remnant cholesterol on carotid IMT, and so future investigations assessing this relationship may be of interest. Additionally, the analysis was conducted at a single centre and the study design did not allow for establishing causal relationships between TG levels and atherosclerosis. Finally, due to substantial differences in age and sex distributions between the controls and HTG groups, statistical adjustment might be insufficient, and the possibility of residual confounding cannot be excluded.

In conclusion, we found that patients with severe HTG do not have significantly increased carotid IMT compared to normal controls once age and sex are accounted for. In contrast, FH patients exhibit significantly greater adjusted carotid IMT than control subjects. These findings remind us of the nuanced role of TG in atherogenesis, particularly in severe HTG, where extreme elevation may not automatically translate into increased carotid IMT. The findings emphasize the complex relationship between TG metabolism and atherosclerosis risk and could prompt future studies addressing this question using different study designs and additional non-invasive imaging modalities, including 2- and 3-dimensional assessment of carotid plaque.

## Declaration of competing interest

The authors declare the following financial interests/personal relationships which may be considered as potential competing interests: Robert A. Hegele reports a relationship with 10.13039/100010875Amgen Canada Inc that includes: consulting or advisory and speaking and lecture fees. Robert A. Hegele reports a relationship with HLS Therapeutics Inc that includes: consulting or advisory and speaking and lecture fees. Robert A. Hegele reports a relationship with 10.13039/100009009Novartis Pharmaceuticals Canada Inc that includes: consulting or advisory and speaking and lecture fees. Robert A. Hegele reports a relationship with 10.13039/100013739Pfizer Canada ULC that includes: consulting or advisory and speaking and lecture fees. Robert A. Hegele reports a relationship with Sanofi-Aventis Canada Inc that includes: consulting or advisory and speaking and lecture fees. Robert A. Hegele reports a relationship with Ionis Pharmaceuticals Inc that includes: consulting or advisory. Robert A. Hegele reports a relationship with Arrowhead Pharmaceuticals Inc that includes: consulting or advisory. The other authors declare that they have no known competing financial interests or personal relationships that could have appeared to influence the work reported in this paper.
